# Use of 3D Printing Techniques to Fabricate Implantable Microelectrodes for Electrochemical Detection of Biomarkers in the Early Diagnosis of Cardiovascular and Neurodegenerative Diseases

**DOI:** 10.1021/acsmeasuresciau.3c00028

**Published:** 2023-09-20

**Authors:** Nemira Zilinskaite, Rajendra P. Shukla, Ausra Baradoke

**Affiliations:** † Wellcome/Cancer Research UK Gurdon Institute, Henry Wellcome Building of Cancer and Developmental Biology, University of Cambridge, Tennis Court Road, Cambridge CB2 1QN, U.K.; ‡ Faculty of Medicine, University of Vilnius, M. K. Čiurlionio g. 21, LT-03101 Vilnius, Lithuania; § BIOS Lab-on-a-Chip Group, MESA+ Institute for Nanotechnology, Max Planck Center for Complex Fluid Dynamics, University of Twente, P.O. Box 217, 7500 AE Enschede, The Netherlands; # Center for Physical Sciences and Technology, Savanoriu 231, LT-02300 Vilnius, Lithuania

**Keywords:** 3D printing, Implantable microelectrodes, Cardiovascular and neurodegenerative diseases, Electrochemical detection, Early diagnosis, Personalized treatment, Stereolithography, Biomarkers

## Abstract

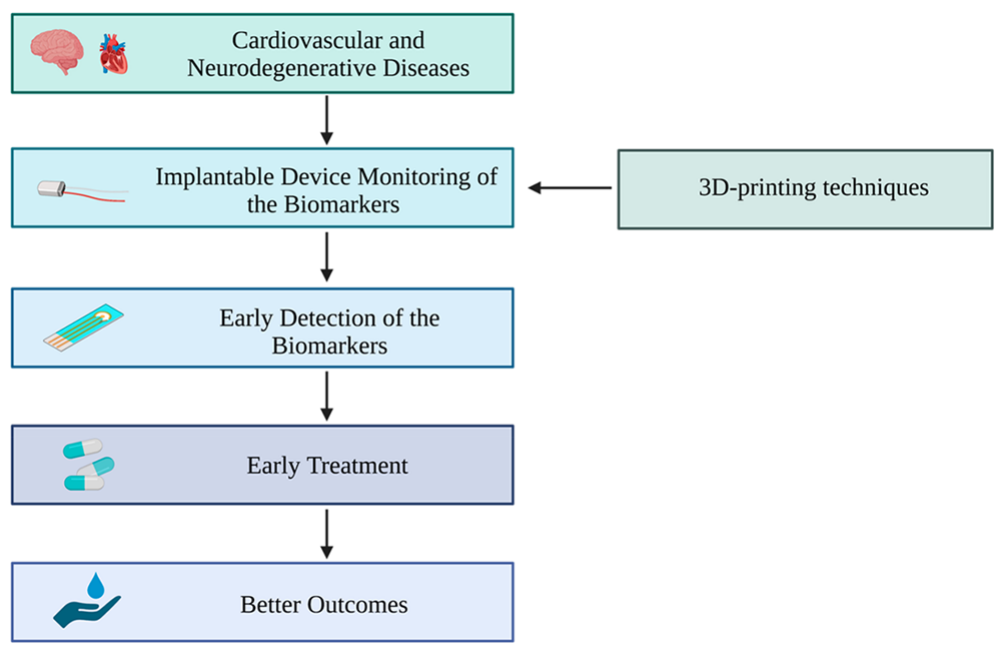

This Review provides a comprehensive overview of 3D printing techniques to fabricate implantable microelectrodes for the electrochemical detection of biomarkers in the early diagnosis of cardiovascular and neurodegenerative diseases. Early diagnosis of these diseases is crucial to improving patient outcomes and reducing healthcare systems' burden. Biomarkers serve as measurable indicators of these diseases, and implantable microelectrodes offer a promising tool for their electrochemical detection. Here, we discuss various 3D printing techniques, including stereolithography (SLA), digital light processing (DLP), fused deposition modeling (FDM), selective laser sintering (SLS), and two-photon polymerization (2PP), highlighting their advantages and limitations in microelectrode fabrication. We also explore the materials used in constructing implantable microelectrodes, emphasizing their biocompatibility and biodegradation properties. The principles of electrochemical detection and the types of sensors utilized are examined, with a focus on their applications in detecting biomarkers for cardiovascular and neurodegenerative diseases. Finally, we address the current challenges and future perspectives in the field of 3D-printed implantable microelectrodes, emphasizing their potential for improving early diagnosis and personalized treatment strategies.

## 1 Introduction

Cardiovascular and neurodegenerative diseases represent significant global health challenges, with early diagnosis and intervention being critical for reducing morbidity and mortality.^1,2^ Conventional diagnostic techniques often detect late-stage symptoms using noninvasive methods, limiting their effectiveness in identifying these diseases during their early stages.^3−5^ One common type of noninvasive microelectrode is the electrocardiogram (ECG) electrode, which measures the heart’s electrical activity. Another type is the electroencephalogram (EEG) electrode, which measures the brain’s electrical activity.^6,7^ Glucose sensors monitor blood glucose levels in patients with diabetes.^8^ Similarly, pH sensors can monitor the acidity or alkalinity of a patient’s blood or other bodily fluids.^9^

Implantable microelectrodes have emerged as a promising tool for research and clinical applications in neurodegenerative and cardiovascular diseases. The development of implantable microelectrodes for the electrochemical detection of biomarkers offers a good alternative, enabling real-time monitoring and early diagnosis of cardiovascular disease (CVD) and neurodegenerative diseases with minimal invasiveness.^10,11^ These devices enable precise and targeted stimulation and monitoring of neural and cardiac activity, allowing for a deeper understanding of the underlying mechanisms of these diseases and therefore informing physicians about potential diagnoses and necessary treatments. With their potential to improve disease management and treatment outcomes, implantable microelectrodes represent a significant advancement in neurology and cardiology.^12^ Further research is needed to optimize these devices’ design and functionality and fully realize their therapeutic potential.

CVD is a leading cause of death worldwide, taking over 17 million lives yearly. CVD encompasses coronary artery disease, heart failure, arrhythmias, and disorders of blood and vessels, among many more. The risk of CVD increases with age and risk factors such as obesity, smoking, physical inactivity, and alcohol abuse.^13^ Early diagnosis by a physician and intervention are crucial for preventing disease progression and optimizing patient outcomes (Table 1).

**Table 1:** List of Biomarkers for Cardiovascular and Neurodegenerative Diseases with Specific Health Issues with References

biomarker	health issue	ref
troponin	acute coronary syndrome	(14)
B-type natriuretic peptide (BNP)	heart failure	(15)
C-reactive protein (CRP)	atherosclerosis	(16)
lipoprotein-associated phospholipase A2	atherosclerosis	(17)
galectin-3	heart failure	(18)
brain natriuretic peptide (BNP)	stroke	(19)
matrix metalloproteinase (MMP)	atherosclerosis	(20)
myeloperoxidase (MPO)	atherosclerosis	(21)
fibrinogen	atherosclerosis	(22)
homocysteine	atherosclerosis	(23)
alpha-synuclein	Parkinson’s disease	(24)
beta-amyloid	Alzheimer’s disease	(25)
tau protein	Alzheimer’s disease	(26)
neurofilament light chain protein (NfL)	multiple sclerosis and other	(27)
amyloid precursor protein (APP)	traumatic brain injury	(28)
glial fibrillary acidic protein (GFAP)	traumatic brain injury	(29)
S100B protein	traumatic brain injury	(30)
UCH-L1 protein	traumatic brain injury	(31)
neurogranin	Alzheimer’s disease	(32)
apolipoprotein E (ApoE)	Alzheimer’s disease	(33)

Neurodegenerative diseases (NDD), such as Alzheimer’s disease (AD) and Parkinson’s disease (PD), are characterized by the progressive loss of neuronal function and structure, resulting in cognitive and motor impairments. Distinguishing between these two diseases is extremely difficult. Therefore, effective biomarkers and applications are essential for early diagnosis.^34,35^ One well-known biomarker for AD is β-amyloid peptide (Aβ), which aggregates into brain plaques.^36^ These plaques have a critical role in the development of the disease, and their detection in cerebrospinal fluid or brain tissue is indicative of AD pathology.^37^ Aβ is involved with neural connection disruption, synaptic dysfunction, and neuronal death in specific brain regions and is a classic prognostic biomarker identifying the potential progression of the disease.^38,39^ Another biomarker for AD is tau protein, which forms neurofibrillary tangles in the brain, contributing to neuronal dysfunction and death (Table 1). Tau protein is involved in microtubule organization, which is necessary for neuronal function. Therefore, protein pathological modification is identified as a hallmark of AD.^40^ The early detection of these biomarkers is essential support for physicians making the final diagnosis of the diseases.

## 2 BIOMARKERS AND CONVENTIONAL DIAGNOSTICS TECHNIQUES IN CARDIOVASCULAR AND NEURODEGENERATIVE DISEASES

### 2.1 Importance of Early Diagnosis

**Figure 1:**
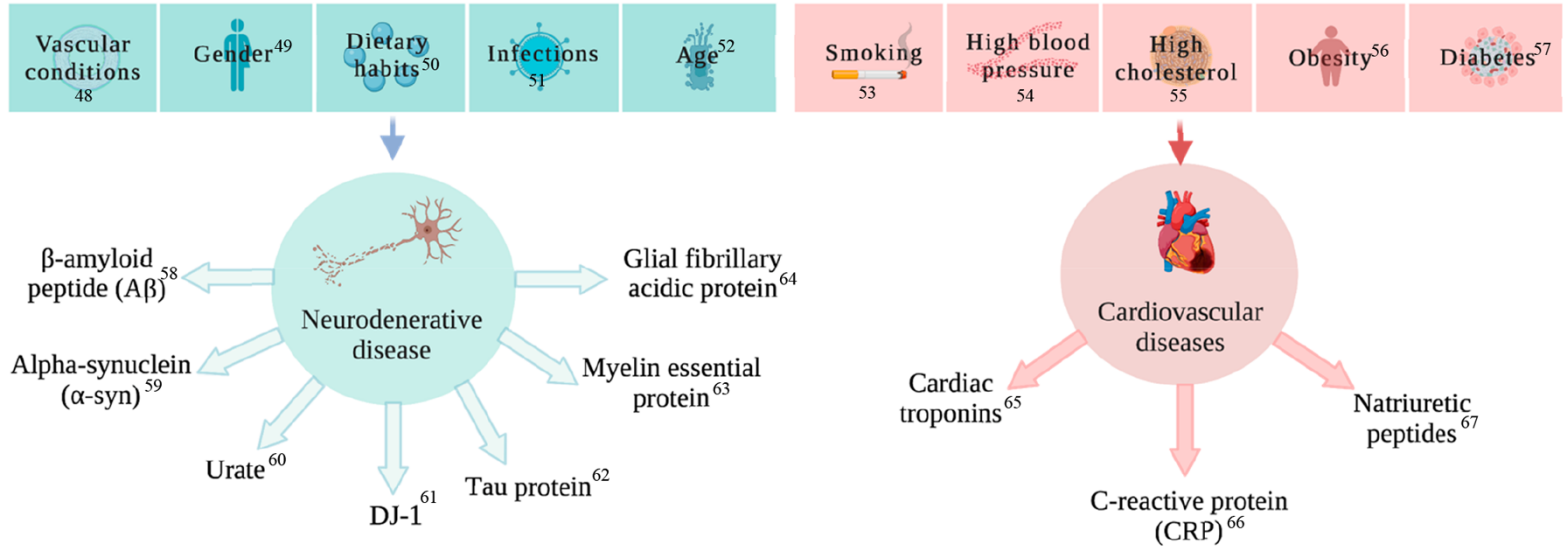
Overview of neurodegenerative and cardiovascular diseases. Schematic diagram illustrating the pathophysiology of common cardiovascular and neurodegenerative diseases, their shared risk factors, and the impact on patients and healthcare systems.^48−67^ (Created with BioRender.com.)

Early diagnosis of cardiovascular and neurodegenerative diseases is vital for initiating timely interventions and optimizing treatment outcomes. By detecting these disease biomarkers in their initial stages, healthcare providers can implement preventative measures and personalized treatment plans, reducing disease progression and improving patients’ quality of life. Biomarkers are crucial in early detection, providing quantitative indicators of the disease presence and progression. Complex diseases such as cardiovascular and neurodegenerative diseases require precise and rapid diagnosis by a physician, as any delay can be detrimental to the patients.^41^ The advancements in the molecular biomarker field and their network allowed for more accurate and robust diagnosis making the implantable microelectrode field advance toward early detection of these diseases (Figure 1).^42^

In cardiovascular diseases such as coronary artery disease and heart failure, early detection enables initiating appropriate therapies and lifestyle changes to prevent disease progression and reduce the risk of adverse events such as myocardial infarction and stroke.^43^ For example, early identification of patients with high blood pressure and dyslipidemia enables the initiation of antihypertensive and lipid-lowering medications, respectively, which have been shown to reduce the risk of cardiovascular events.^44^ In addition, early diagnosis of heart failure can lead to the initiation of guideline-directed medical therapies, such as angiotensin-converting enzyme inhibitors and beta-blockers, which can improve symptoms and reduce the risk of hospitalization and mortality.^45^

Similarly, in neurodegenerative diseases such as AD and PD, early diagnosis enables the initiation of disease-modifying therapies and lifestyle changes to slow disease progression and improve quality of life.^46,47^ For example, early diagnosis of Alzheimer’s enables the initiation of cholinesterase inhibitors, which can enhance cognitive function.^46^ In addition, early diagnosis of PD can lead to the initiation of dopamine replacement therapies, which can improve motor symptoms and the quality of life. Overall, early diagnosis is critical in cardiovascular and neurodegenerative diseases, as it can lead to better treatment outcomes and improved quality of life for patients (Figure 1).

### 2.2 Biomarkers in Cardiovascular Disease

Biomarkers are “characteristics that are objectively measured and evaluated as indicators of normal biological processes, pathogenic processes, or pharmacologic responses to a therapeutic intervention.”^68^ Biomarker detection has revolutionized CVD diagnostics, providing a fast and accurate diagnosis of patients.^69^ These biomarkers are measurable in various biological fluids, including blood, urine, and cerebrospinal fluid, and can provide quantitative indicators of the disease presence and severity. Several biomarkers have been studied and employed for diagnostic purposes in CVD, including cardiac troponins, C-reactive protein (CRP), and natriuretic peptides (Table 2).

**Table 2:** Examples of Biomarkers for Early Diagnosis of Cardiovascular Diseases

serial no.	biomarker type	diagnostic test	test type	sample type	technique	sensitivity	limit of detection	sample volume	test time	Cost	ref
1	troponin	high-sensitivity troponin assay	blood test	serum or plasma	immunoassay	90–97%	1–2 ng/L	1 mL	within 1–3 h of symptoms	$15–$300	(14, 70−73)
2	B-type natriuretic peptide (BNP)	BNP blood test	blood test	serum or plasma	immunoassay	90–95%	5 pg/mL	1 mL	same day	$50–$150	(74−79)
3	C-reactive protein (CRP)	high-sensitivity CRP assay	blood test	serum or plasma	immunoassay	80–90%	0.1 mg/L	1 mL	same day	$20–$100	(80−84)
4	myeloperoxidase (MPO)	MPO blood test	blood test	serum or plasma	immunoassay	70–85%	1 ng/mL	1 mL	same day	$100–$150	(85−87)
5	lipoprotein-associated phospholipase A2 (Lp-PLA2)	Lp-PLA2 blood test | Blood test	blood test	serum or plasma	immunoassay	60–80%	15 ng/mL	1 mL	same day	$75–$150	(88−90)
6	soluble urokinase plasminogen activator receptor (suPAR)	suPARnostic ELISA	blood test	serum or plasma	ELISA	65–75%	1.8 ng/mL	100 μL	same day	$70–$100	(91−93)
7	matrix metalloproteinase-9 (MMP-9)	MMP-9 blood test	blood test	serum or plasma	immunoassay	75–80%	1 ng/mL	1 mL	same day	$100–$150	(94−96)
8	fibrinogen	fibrinogen blood test	blood test	serum or plasma	coagulation assay	80–90%	100 mg/dL	1 mL	same day	$20–$100	(97−99)
9	homocysteine	homocysteine blood test	blood test	serum or plasma	immunoassay	75–85%	1 μmol/L	1 mL	same day	$30–$80	(100−102)

Cardiac troponins are a group of structural proteins specific to actin filaments of striated cardiac muscles. Troponins are an old standard biomarker for acute myocardial infarction (AMI), and their detection and quantification have been critical in the early management of AMI patients.^103^ Additionally, elevated levels of cardiac troponins have been associated with worse outcomes in patients with heart failure, pulmonary embolism, and other cardiac conditions.^104^

CRP is an acute-phase protein synthesized by the liver in response to inflammation.^105^ CRP binds to lysophosphatidylcholine on dead cells and bacteria, activating the complement pathway.^106^ It has been shown that elevated levels of CRP have been linked to increased cardiovascular risk and have been proposed as a biomarker for predicting and diagnosing CVD.^107^ CRP can also monitor disease progression and treatment response in patients with atherosclerosis and other inflammatory.^108−110^

Natriuretic peptides are hormone-like molecules secreted by the heart in response to increased left ventricular wall stress and stretch. Brain natriuretic peptide (BNP) and N-terminal pro-BNP (NT-proBNP) are both biomarkers for heart failure and are clinically used to detect heart dysfunction^111^ Mechanical stretch is mainly caused by the BNP rise by myocardium, although the exact mechanism remains unclear. Elevated levels of natriuretic peptides have been associated with increased cardiovascular risk and are proposed as biomarkers for diagnosing and managing heart failure and other cardiac conditions. BNP and NT-proBNP levels were elevated in AMI, atrial fibrillation, and cardiomyopathies, indicating the promising role of natriuretic peptides as biosensors for these disorders.^112−118^

Biomarkers can help distinguish between stages or subtypes of cardiovascular diseases by reflecting various aspects of disease progression, severity, and underlying mechanisms. For instance, in heart failure, biomarkers like B-type BNP and N-terminal pro-BNP (NT-proBNP) can indicate the degree of cardiac strain and help categorize heart failure into different stages.^119,120^ Troponin levels are crucial for diagnosing and differentiating between different types of myocardial infarction.^121^ High-sensitivity C-reactive protein (hs-CRP) and interleukin-6 (IL-6) assess inflammation, a critical factor in atherosclerosis and coronary artery disease.^122^ These diagnoses are performed by physicians, who can evaluate other symptoms related to the condition to make an accurate diagnosis.

Biomarkers can help monitor disease progression and treatment response in cardiovascular diseases by reflecting changes in cardiac function, inflammation, and tissue damage.^123^ For instance, in heart failure, monitoring BNP and NT-proBNP levels over time can help assess changes in cardiac strain and the effectiveness of medications.^124^ Troponin levels are commonly monitored to track myocardial damage in acute coronary syndrome and evaluate the success of interventions like angioplasty.^125^ These indications can help physicians make the correct diagnoses and treat patients accordingly.

### 2.3 Biomarkers in Neurodegenerative Disease

In Parkinson’s disease, the protein α-synuclein (α-syn) has been identified as a critical biomarker. This protein aggregates into Lewy bodies in the brain, which are associated with the degeneration of dopaminergic neurons and the development of motor symptoms.^126^ Clinical diagnosis generally occurs too late due to early loss of nigral neurons, and only then do symptoms show as motor neuron degeneration begins.^127^ Late diagnosis does not allow for neuroprotective treatments; therefore, an early diagnosis is necessary. Other biomarkers for PD include urate, a molecule involved in antioxidant defense. Urate is thought to protect dopaminergic neurons from degeneration and, therefore, can be suitable for early diagnosis due to elevated levels in Parkinson’s patients^128−130^ Generally, elevated urate levels in healthy individuals are associated with reduced risk for PD.^131−133^ It is essential to mention that urate is also a biomarker for other diseases such as chronic gout. Therefore, diagnosis using this marker should be performed with other markers.^134^ Another biomarker of PD is DJ-1, a protein that regulates cellular stress responses. DJ-1 is thought to have neuroprotective properties and protect dopaminergic neurons while acting as an oxidative stress sensor and antioxidant (Table 3).

**Table 3:** Examples of Biomarkers for Early Diagnosis of Neurodegenerative Diseases

biomarker	biomarker type	diagnostic test	sample type	technique	sensitivity	limit of detection	sample volume	test time	cost	ref
tau	protein	ELISA	cerebrospinal fluid	immunoassay	90–100%	0.013 ng/mL	150 μL	4 h	$300–$500	(135, 136)
beta-amyloid 42	protein	ELISA	cerebrospinal fluid	immunoassay	80–90%	23.9 pg/mL	200 μL	4–6 h	$250–$400	(137, 138)
alpha-synuclein	protein	ELISA	cerebrospinal fluid	immunoassay	83–96%	0.02 ng/mL	200 μL	5 h	$350–$500	(127, 139−141)
neurofilament light chain	protein	ELISA	blood	immunoassay	90–100%	5.1 pg/mL	200 μL	2–3 h	$150–$300	(142−145)
glial fibrillary acidic protein	protein	ELISA	blood	immunoassay	80–95%	2.4 ng/mL	200 μL	4 h	$200–$400	(146, 147)
ubiquitin C-terminal hydrolase L1	protein	ELISA	blood	immunoassay	85–95%	0.005 ng/mL	100 μL	2 h	$200–$300	(148, 149)
amyloid beta 1–40	protein	ELISA	cerebrospinal fluid	immunoassay	80–90%	10 pg/mL	200 μL	4–6 h	$250–$400	(150, 151)
phosphorylated tau	protein	ELISA	cerebrospinal fluid	immunoassay	80–90%	0.8 pg/mL	150 μL	4 h	$300–$500	(152, 153)
total tau	protein	ELISA	blood	immunoassay	90–100%	4.4 pg/mL	200 μL	3–4 h	$150–$300	(154, 155)
neurogranin	protein	ELISA	cerebrospinal fluid	immunoassay	80–90%	0.1 ng/mL	200 μL	5–6 h	$350–$500	(156, 157)
chitinase-3-like protein 1	protein	ELISA	blood	immunoassay	80–90%	0.12 ng/mL	200 μL	3–4 h	$200–$400	(158−160)
beta-secretase 1	protein	ELISA	cerebrospinal fluid	immunoassay	85–95%	0.5 pg/mL	200 μL	5 h	$300–$500	(161, 162)

Recent studies have also identified potential biomarkers for other neurodegenerative diseases, such as amyotrophic lateral sclerosis (ALS), Huntington’s disease, and multiple sclerosis^163−165^ These biomarkers include glial fibrillary acidic protein and myelin essential protein, which are indicative of neuronal damage, inflammation, and demyelination, respectively^166,167^

Identifying and detecting these biomarkers provide valuable insights into the underlying pathophysiology of neurodegenerative diseases, enabling earlier diagnosis and personalized treatment strategies.^168^ However, challenges remain in developing accurate and reliable detection methods, particularly for low-abundance biomarkers in the early stages of the disease. Using 3D-printed implantable microelectrodes for electrochemical detection of these biomarkers offers a promising approach for addressing these challenges, enabling real-time, in vivo monitoring of biomarker levels.^169^

Biomarkers are valuable tools for distinguishing between stages or subtypes of neurodegenerative diseases by reflecting specific pathological changes in the brain.^170,171^ In AD, biomarkers like beta-amyloid and tau proteins in cerebrospinal fluid or imaging scans can help differentiate between different stages of disease progression.^172,173^ For Parkinson’s disease, levels of alpha-synuclein or dopamine in various bodily fluids can indicate disease severity.^174,175^ In multiple sclerosis, biomarkers such as myelin basic protein (MBP) and neurofilament light chain (NfL) can differentiate between relapsing-remitting and progressive forms of the disease. The physician considers all of the symptoms and evaluates the biomarker presence to make an informed diagnosis about the patient’s progression.

In neurodegenerative diseases, biomarkers are crucial for monitoring disease progression and the response to treatment. For Alzheimer’s disease, tracking levels of beta-amyloid and tau proteins can indicate the accumulation of pathological changes in the brain.^176−178^ Neurofilament light chain levels can be used to assess neuronal damage in various neurodegenerative conditions, including multiple sclerosis.^179^ Monitoring biomarkers like alpha-synuclein in Parkinson’s disease helps gauge disease progression and evaluate the effectiveness of therapies.^180^

### 2.4 Limitations of Conventional Diagnostic Techniques

Conventional diagnostic techniques for cardiovascular and neurodegenerative diseases, such as blood tests, imaging, and clinical assessments, have advantages and limitations that are important to address. Many conventional diagnostic techniques lack the sensitivity and specificity required for the early detection and accurate diagnosis of cardiovascular and neurodegenerative diseases, which implantable microelectrodes can improve.

Blood tests for cardiac biomarkers, such as troponin and BNP, are highly regarded in the medical field due to their exceptional diagnostic and prognostic value in assessing various aspects of cardiac health and function. These tests provide crucial insights into cardiovascular conditions and play a pivotal role in acute and chronic settings. However, troponin or BNP may not be elevated until several hours after the onset of symptoms, limiting their usefulness for early diagnosis.^181^ This could be addressed by using implantable microelectrodes that monitor biomarker levels constantly, providing information about disease progression.

Magnetic resonance imaging (MRI) and computerized tomography (CT) are excellent imaging techniques for diagnosing brain damage because they provide detailed, noninvasive, multidimensional, and contrast-enhanced images. Their ability to differentiate between tissue types and reveal functional information further enhances their diagnostic utility.^182,183^ These imaging methods are pivotal in guiding medical decision-making, treatment planning, and patient management for individuals with brain injuries or disorders. However, these techniques may not detect subtle changes in brain structure and function in the early stages of neurodegenerative diseases, resulting in delayed diagnosis and suboptimal treatment outcomes.^184^ Implantable microelectrodes could be helpful for physicians to avoid these limitations and track subtle changes.

Cardiac catheterization is a minimally invasive and precise procedure that provides real-time visualization of the heart’s structures and blood vessels. It offers accurate diagnosis, therapeutic interventions, and rapid recovery for heart conditions, including coronary artery disease and valve abnormalities. With reduced risks, shorter recovery times, and the potential for same-day procedures, cardiac catheterization improves patient outcomes and quality of life.^185^ This approach continues to drive cardiovascular research and technology advancements, making it a valuable tool in modern cardiology. However, this technique involves the insertion of a catheter into the heart, which carries a risk of complications, such as bleeding, infection, or stroke. An implantable microelectrode could minimize catheterization usage, limit complications, and improve patient outcomes.

Positron emission tomography (PET) imaging offers numerous advantages in medical diagnostics. It provides unparalleled insights into molecular and metabolic processes within the body, aiding in disease detection and precise treatment planning.^186^ PET’s ability to visualize cellular activity and receptor interactions at a molecular level enables accurate diagnosis and staging of various conditions, including cancer, neurological disorders, and cardiovascular diseases. However, this technique requires the injection of a radioactive tracer, which can be costly and pose potential health risks to patients.^187^ PET also requires specialized equipment, which might not be available in smaller and more remote hospitals, leaving some patients without proper diagnosis. Microelectrodes implanted into the body could minimize PET use and improve access to the correct treatment, even in remote areas.

Stress tests and electroencephalograms (EEGs) offer unique medical diagnostics advantages. Stress tests, such as exercise or pharmacological stress tests, provide valuable insights into cardiovascular health by assessing the heart’s response to physical exertion or medication. These tests help detect coronary artery disease, evaluate exercise capacity, and determine the presence of heart rhythm abnormalities.^188,189^ On the other hand, EEGs record electrical brain activity, aiding in diagnosing and monitoring various neurological conditions including epilepsy, sleep disorders, and brain injuries. EEGs provide real-time brain function data and allow for the identification of abnormal patterns or seizure activity. Both stress tests and EEGs play essential roles in different medical domains, contributing to improved patient care and tailored treatment strategies. It is vital to mention that stress tests, or EEGs, may require patients to undergo uncomfortable or stressful procedures, reducing patient compliance and data quality.^190^ Stress tests involving physical exertion to assess cardiac function may not be feasible for elderly or frail patients, limiting their usefulness in these populations. Similarly, EEGs, which involve the placement of electrodes on the scalp to measure brain activity, may be uncomfortable or claustrophobic for some patients, leading to incomplete or inaccurate data.^191^ The limitations could be avoided by implantable microelectrodes, preventing frail patients from undergoing these tests and improving their overall mental health and data quality.

The limitations of conventional diagnostic techniques highlight the need for innovative, noninvasive, and dynamic monitoring approaches for the early detection and personalized treatment of cardiovascular and neurodegenerative diseases. 3D-printed implantable microelectrodes for electrochemical detection of biomarkers offer a promising approach to overcome these limitations, enabling real-time, in vivo monitoring of disease biomarkers with high sensitivity, selectivity, and patient comfort.

## 3 ELECTROCHEMICAL DETECTION OF BIOMARKERS FOR THE EARLY DIAGNOSIS OF CVD AND NDD: ADVANTAGES AND LIMITATIONS

### 3.1 Principles of Electrochemical Detection

**Figure 2:**
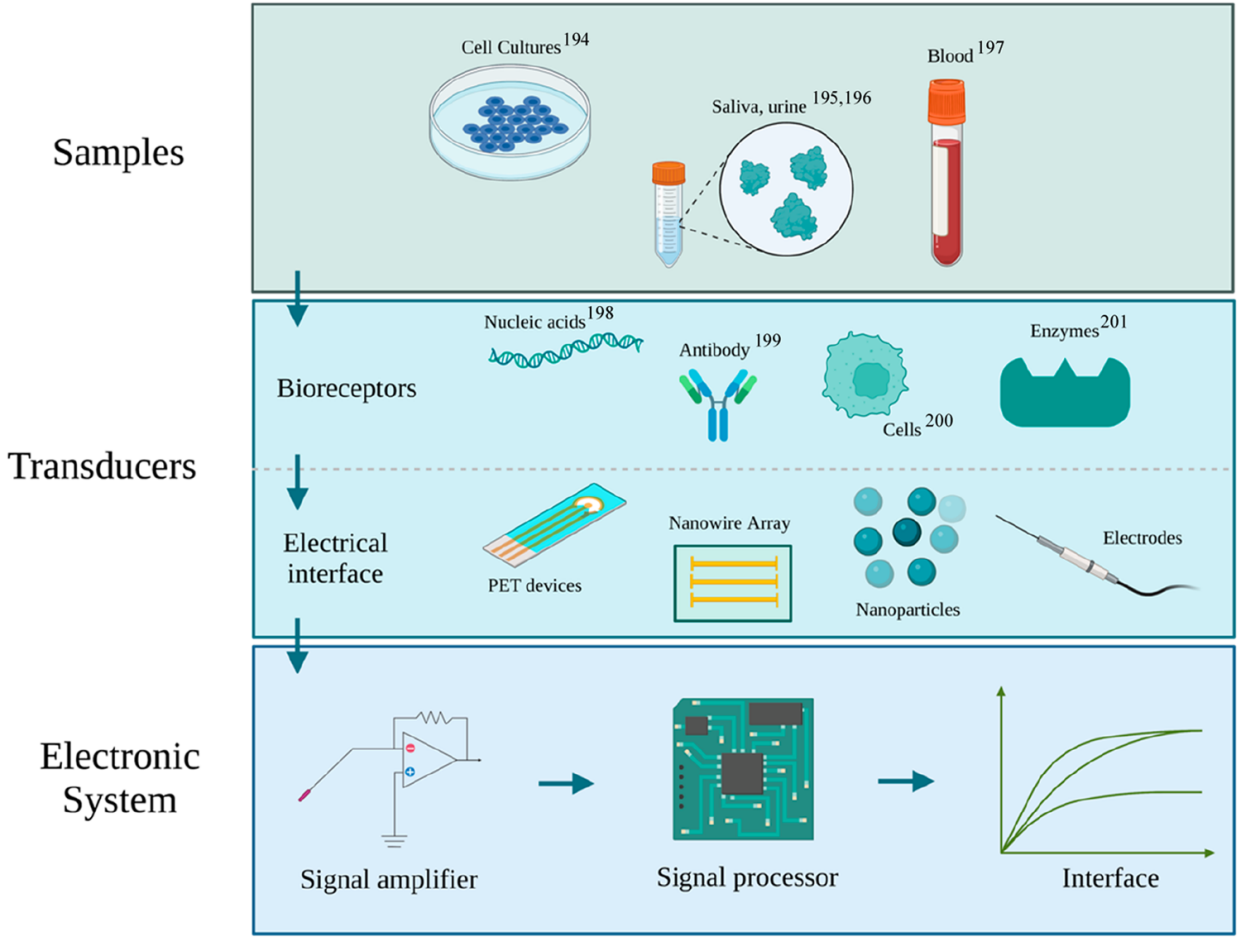
Electrochemical detection principle starts from samples including cell culture, blood, saliva, or urine. Bioreceptors can detect them, and the signal can be transduced via an electrical interface to a signal amplifier. Then the signal is processed, and electrochemical detection graphs are produced for analysis.^194−201^ Adapted with permission under a Creative Commons License from ref (202). Copyright 2008, Sensors.

Electrochemical detection is based on the measurement of electrical signals generated by redox reactions between the target biomarker and a recognition element immobilized on the surface of an electrode. The electrical signals, which are proportional to the concentration of the target biomarker, can be quantified using various electrochemical techniques, such as amperometry, voltammetry, or impedance spectroscopy.^192^ The electrochemical detection test’s selectivity and sensitivity can be significantly enhanced by optimizing the electrode’s design and surface functionalization. This enables the accurate measurement of low-abundance biomarkers in complex biological samples.^193^ In the following sections, we discuss how 3D printing techniques have been employed to fabricate implantable microelectrodes for the electrochemical detection of biomarkers, addressing critical design considerations and exploring their potential applications in diagnosing cardiovascular and neurodegenerative diseases (Figure 2).

### 3.2 Advantages of Electrochemical Detection

**Figure 3:**
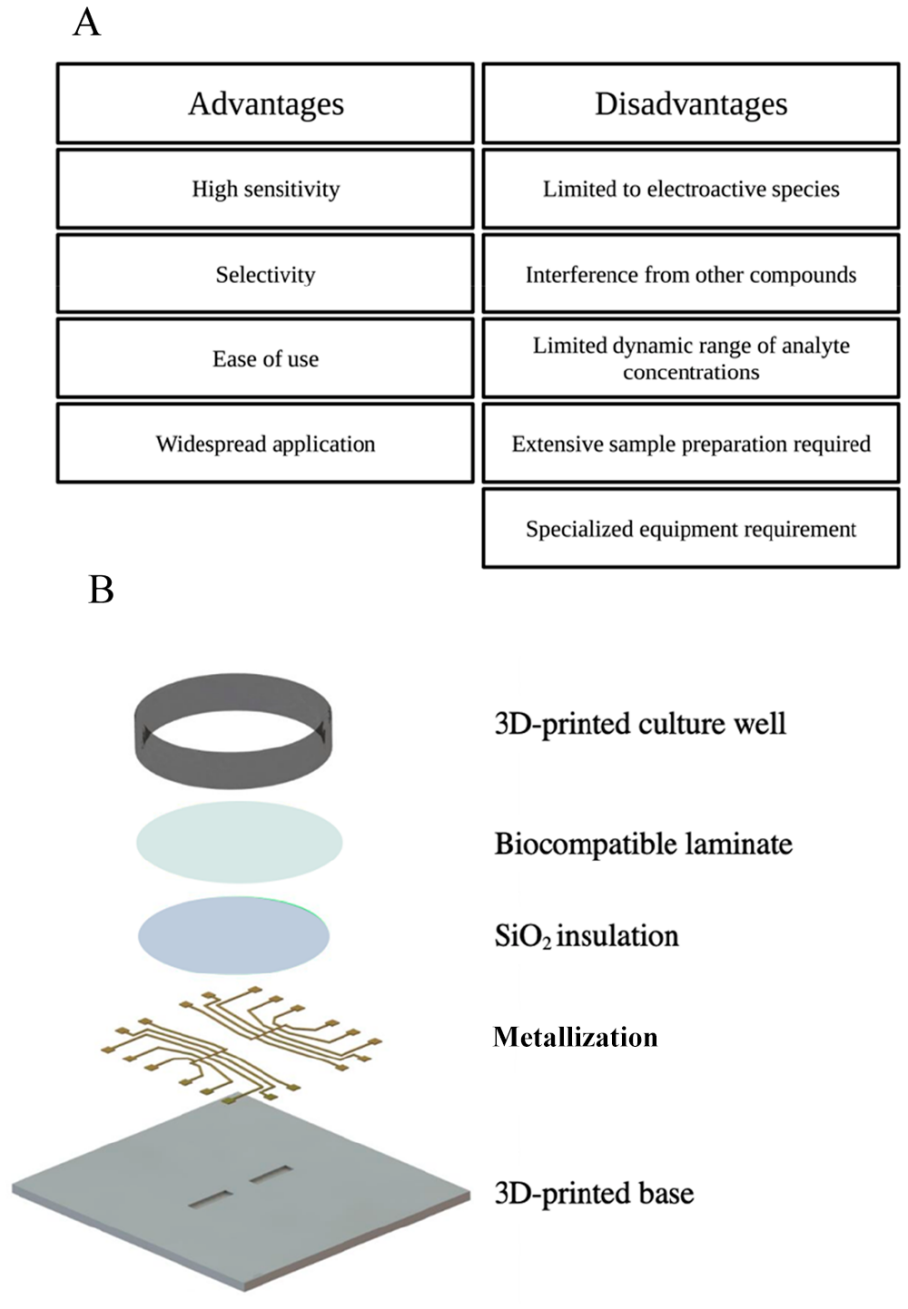
(A) Summary of advantages and disadvantages of electrochemical detection. B) Schematic of a 3D-printed implantable microelectrode. Adapted from ref (210). Copyright 2021 American Chemical Society.

Electrochemical detection of biomarkers presents several advantages over traditional diagnostic techniques. First, electrochemical biosensors have high sensitivity, enabling the detection of low biomarker concentrations in various sample types^203^ electrochemical biosensors exhibit rapid response times, enabling real-time monitoring of biomarker levels. Third, electrochemical biosensors can be miniaturized and integrated into implantable devices, enabling tracking of biomarker levels and facilitating personalized treatment plans.^204^ These features make electrochemical detection a promising approach for the early diagnosis of cardiovascular and neurodegenerative diseases. By enabling real-time, in vivo monitoring of biomarker levels, electrochemical biosensors can provide clinicians with valuable information for developing personalized treatment plans. Additionally, the high sensitivity of electrochemical biosensors may enable the detection of disease biomarkers in the early stages of disease development before symptoms become apparent (Figure 3A).

### 3.3 Limitations of Electrochemical Detection

Due to its high sensitivity, selectivity, and ease of use, electrochemical detection has become widespread in many fields, including analytical chemistry, biosensors, and medical diagnostics. However, several limitations of electrochemical detection should be taken into consideration. Electrochemical detection is primarily used to analyze electroactive species, compounds that can undergo oxidation or reduction at an electrode. Therefore, it is not suitable for the detection of nonelectroactive species.^205^ Other compounds in the sample can affect it, interfering with the detection of the analyte of interest. Electrochemical detection has a limited dynamic range of analyte concentrations over which the detector can provide accurate measurements.^206^

Electrochemical detection often requires extensive sample preparation, such as adding reagents or extracting the analyte from the sample matrix. This can be time-consuming and may also introduce additional sources of error. Electrodes used in electrochemical detection can become fouled over time, reducing their sensitivity and increasing their noise level. This can be a problem when analyzing complex samples or samples with high levels of interfering compounds. Electrochemical detection typically requires specialized equipment, such as a potentiostat or electrochemical cell, which can limit its portability and ease of use in the field or at the point of care (Figure 3A).

While electrochemical detection is a powerful analytical technique with many advantages, its limitations should be considered when selecting an appropriate detection method for a particular application.

## 4 3D Printing Techniques for Fabricating Implantable Microelectrodes

### 4.1 Overview of 3D Printing Technologies

3D printing, also known as additive manufacturing, is a rapidly advancing technology that enables the fabrication of three-dimensional objects with complex geometries and features.^207^ Various 3D printing techniques have been developed, including stereolithography (SLA), selective laser sintering (SLS), fused deposition modeling (FDM), and inkjet-based printing. These techniques differ in materials, processing methods, and resolutions, but all share the principle of creating objects layer-by-layer through controlled material deposition.

### 4.2 Advantages of 3D Printing for Implantable Microelectrode Fabrication

3D printing offers several advantages for fabricating implantable microelectrodes, including design flexibility, rapid prototyping, scalability, and customization. With 3D printing, researchers can create microelectrodes with intricate geometries and features, optimizing their performance for specific applications.^208^ Additionally, 3D printing allows for rapid prototyping and iteration of designs, accelerating development and reducing costs associated with traditional manufacturing methods. Furthermore, 3D printing enables the fabrication of patient-specific implantable microelectrodes tailored to individual needs, enhancing their biocompatibility and clinical effectiveness (Figure 3B).^209^

### 4.3 Selection of 3D Printing Techniques for Implantable Microelectrodes

Medical technology has witnessed remarkable advancements in integrating 3D printing techniques into the fabrication of implantable microelectrodes. These miniature devices hold immense potential for applications ranging from neural interfaces to biosensors, promising to revolutionize healthcare. A pivotal decision in this process is the selection of the appropriate 3D printing techniques and materials. The choice of a 3D printing technique profoundly influences implantable microelectrodes’ precision, complexity, and material compatibility. Table 4 summarizes different 3D printing techniques offering distinct advantages and challenges.

**Table 4:** Comparison of 3D Printing Techniques for Fabrication of an Implantable Microelectrode

serial no.	technique	process	advantages	limitations	materials	accuracy	speed	applications	refs
1	FDM (fused deposition modeling)	a nozzle melts and extrudes thermoplastic filament layer by layer	low cost, high speed	limited resolution, poor surface finish	thermoplastics, hydrogels	moderate to high	slow to moderate	prototyping, tooling, functional parts, architectural models	(218−222)
2	SLA (stereolithography)	a laser is used to solidify liquid resin layer by layer	high resolution, good surface finish	limited material choices require postprocessing	resins, plastics, ceramics, and metals.	high (up to 0.01 mm)	moderate to fast	prototyping, dental and medical models, small-scale production	(223−225)
3	SLS (selective laser sintering)	a laser is used to fuse powdered material layer by layer	wide range of materials, good mechanical properties	limited resolution requires postprocessing	metals, ceramics, polymers	high (up to 0.1 mm)	moderate to fast	prototyping, tooling, functional parts, aerospace, and automotive parts	(226−229)
5	DLP (digital light processing)	a light projector shines UV light onto a vat of photosensitive resin, solidifying it layer by layer	high resolution, good surface finish	limited material choices require postprocessing	photopolymers	high (up to 0.01 mm)	fast	prototyping, dental and medical models, small-scale production	(230−232)
6	two-photon polymerization (2PP)	a laser is used to polymerize a photosensitive resin, creating intricate structures	high resolution, ability to create complex structures	limited build volume, slow speed, expensive	photopolymers and hydrogels.	ultrahigh (down to 10 nm)	slow	microfluidics, biomedical devices, micro-optics	(233−235)

Stereolithography (SLA) employs a UV laser to solidify liquid resin layer by layer, resulting in high-resolution, intricate structures. SLA’s ability to create complex structures with microscopic precision is a hallmark of its applicability in implantable microelectrodes. The UV laser selectively solidifies liquid photopolymer resins layer by layer. This precise control enables the fabrication of intricate electrode designs, crucial for accurate neural interfaces or biosensors. SLA excels in producing patient-specific designs, allowing for customization that enhances device-tissue interaction and minimizes foreign body responses. Tailoring the geometry and surface properties of the microelectrode contributes to improved biocompatibility and performance. SLA produces microelectrodes with smooth surfaces, reducing the risk of tissue irritation and inflammation. Moreover, the technique facilitates the incorporation of specialized coatings or surface modifications to enhance tissue integration and minimize the immune response.^211^ This surface engineering capability is vital for creating biocompatible microelectrodes.

SLA poses significant preselection challenges, particularly concerning biocompatibility. Materials used in SLA-printed implantable microelectrodes demand a thorough assessment for compatibility with biological tissues over their operational lifespan. Ensuring successful, long-term implants requires stringent biocompatibility tests to avert adverse reactions. Postprocessing is often necessary to optimize the mechanical properties of SLA-printed components, but this must not compromise material biocompatibility or performance. Conductivity enhancement in SLA-printed materials is a work in progress, necessitating careful consideration of conductivity versus biocompatibility. Extensive research is crucial for refining material formulations to achieve optimal electrical performance. Long-term stability within the body remains a concern for SLA-printed microelectrodes, necessitating comprehensive solutions for material degradation, wear, and corrosion to maintain performance and patient safety over time.^211^

Selective laser sintering (SLS) is a transformative technique in implantable microelectrodes, utilizing a high-energy laser to fuse conductive metals and ceramics. This method’s versatility is a cornerstone, accommodating various materials, including conductive metals and ceramics, promising enhanced electrical conductivity for improved signal quality and data acquisition in vital applications like neural interfaces and biosensors. SLS’s precision in fabricating intricate microelectrode designs and its rapid prototyping capabilities facilitate iterative design and testing, shortening development timelines and reducing costs. Integrating electronics within microelectrodes during the SLS fabrication process further underscores its potential for enhanced functionality and reliability, ushering in a new era of advanced medical devices and patient-centric solutions.^212^

SLS boasts remarkable versatility; however, achieving a resolution comparable to that of microfabrication or photolithography poses a challenge. The potential for surface roughness to impact tissue integration and long-term biocompatibility highlights the need to harmonize resolution with material characteristics for optimal device performance. Biocompatibility is a critical concern for SLS-printed materials, necessitating thorough evaluations due to the potential release of particles during sintering. The material choice significantly influences implant success, demanding the consideration of long-term effects. The layering process in SLS may result in porosity within printed parts, potentially affecting the material stability and tissue integration. Addressing porosity through postprocessing, including sintering optimization and surface treatments, is imperative for overcoming associated challenges and ensuring the functionality of implantable microelectrodes.^212^

Fused deposition modeling (FDM) involves the layered extrusion of molten material, yielding cost-effectiveness and versatility. This technique accommodates diverse biocompatible materials, making it an appealing choice for medical device development due to its cost-effectiveness, enabling swift iteration and microelectrode testing and thus enhancing innovation efficiency. Implantable microelectrodes’ need for anatomical precision finds a solution in FDM’s flexibility, facilitating patient-specific designs that promote device-tissue compatibility and integration.^213^ FDM’s extensive material compatibility, encompassing biocompatible variants, empowers researchers to explore materials aligned with desired electrical and mechanical traits, ultimately enhancing microelectrode functionality and biocompatibility. Notably, FDM excels at seamlessly integrating features within microelectrodes, encompassing drug delivery channels and real-time monitoring sensors, promising multifunctional microelectrodes poised to elevate therapeutic and diagnostic capabilities.^214^ In summary, FDM encapsulates a spectrum of advantages for implantable microelectrodes, amalgamating cost-effectiveness, customization, versatile material adaptability, and multifunctional integration to shape the future landscape of medical technology and patient care.

The layer-by-layer construction inherent in FDM can lead to comparatively lower resolution when contrasted with alternative techniques. This process might also induce surface roughness, potentially influencing tissue integration and biocompatibility. To achieve desired outcomes, meticulous material selection and strategic postprocessing measures become imperative. While FDM can employ biocompatible materials, striking a delicate equilibrium between biocompatibility and electrical conductivity poses a formidable challenge. As implantable microelectrodes contend with diverse mechanical forces within the body, maintaining structural integrity and longevity in FDM-printed microelectrodes hinges on a comprehensive understanding of the material dynamics and device design. The significance of postprocessing steps, including sterilization, cannot be overstated, as they wield the power to impact the final microelectrode’s biocompatibility and mechanical characteristics. Thus, safeguarding the functionality and safety of the device mandates careful postprocessing procedures.^215^

Inkjet printing precisely deposits conductive ink droplets on substrates, allowing for rapid prototyping and biological ink utilization. While suitable for specific tasks, achieving high resolution and uniformity remains challenging. It offers exceptional precision for crafting intricate microelectrode designs, which is crucial for customizing configurations to match individual anatomies and enhancing device compatibility and integration. The method’s versatility spans diverse materials, including conductive polymers and biocompatible substances, empowering microelectrode designs with optimized electrical and mechanical properties. Layer-by-layer deposition supports complex multilayer structures, integrating sensors, stimulators, and drug delivery systems within a single device, augmenting implantable microelectrode capabilities.^216^ With rapid speed and precision, inkjet printing enables high-throughput fabrication, curtailing production times and costs, thereby expediting innovation translation from lab to clinic.^217^

However, inkjet printing comes with drawbacks. Balancing material properties—conductivity, mechanical strength, and biocompatibility—requires meticulous research. Achieving a high-resolution and smooth surface finish in printed microelectrodes is challenging due to droplet size limitations, necessitating postprocessing to address potential tissue integration issues. Ensuring the stability of inkjet-printed microelectrodes during prolonged implantation is another significant challenge, demanding rigorous testing and optimization.^216^

The optimal combination of 3D printing techniques and materials is pivotal in the fabrication of implantable microelectrodes. The chosen approach impacts device precision, complexity, biocompatibility, and performance. A well-informed decision requires a thorough understanding of the specific application, the interaction between the microelectrode and biological tissues, and the desired device functionality. As research and development in 3D printing technologies and materials continue, the potential for enhancing implantable microelectrodes’ performance, safety, and impact on medical science remains truly exciting.

### 4.4 Improving the Resolution of 3D Printing Techniques

Improving the resolution and precision of 3D printing techniques to create more intricate and accurate implantable microelectrodes involves a combination of technological advancements, material innovation, and process optimization. Each technique can be improved using various methods, although most optimization techniques can be allocated to either improvement of hardware or software.

Improving 3D printing precision often begins with hardware enhancements. Newer technologies, such as high-resolution SLA and digital light processing (DLP), offer finer features and smoother surfaces. Reducing the nozzle size or laser beam width enhances detail. Using specialized materials with improved flow, adhesion, and minimal shrinkage improves accuracy. Optimizing the layer thickness in layer-by-layer printing improves surface smoothness and accuracy. Multimaterial printing integrates diverse materials in one design, elevating functionality and precision. These strategies collectively enhance the precision of 3D-printed microelectrodes.

Software and postprinting enhancements are crucial to optimize 3D printed microelectrode design. Postprocessing methods such as polishing, smoothing, and chemical treatments enhance surface quality, reducing roughness and improving accuracy. Advanced design software and algorithms refine digital models, ensuring an accurate translation during printing. During printing, real-time monitoring and feedback mechanisms identify and rectify deviations, preserving precision. These combined strategies contribute to refined microelectrode designs with heightened accuracy and improved quality.

## 5 Design Considerations for 3D-Printed Implantable Microelectrodes

### 5.1 Biocompatibility

Biocompatibility is a critical factor in the design of implantable microelectrodes, as it directly impacts the safety and long-term performance of the device.^236^ The materials fabricating 3D-printed implantable microelectrodes should be biocompatible, nontoxic, and nonimmunogenic, ensuring minimal adverse reactions when implanted in the body. Additionally, surface modifications, such as coating with biocompatible polymers or bioactive molecules, can further enhance the biocompatibility and integration of the microelectrode with the surrounding tissue^237^

### 5.2 Device Geometry and Configuration

The geometry and configuration of the implantable microelectrode can significantly impact its electrochemical performance, including sensitivity, signal-to-noise ratio, and spatial resolution. 3D printing enables the fabrication of microelectrodes with various geometries, such as planar, cylindrical, or needle-like structures, allowing researchers to optimize the device design for specific applications.^238^ Additionally, the arrangement and spacing of the electrode sites can be tailored to enhance the detection of target biomarkers and minimize interference from nontarget species.

### 5.3 Surface Functionalization and Recognition Elements

Surface functionalization of the implantable microelectrode is essential for enhancing its selectivity and sensitivity toward the target biomarkers. Various recognition elements, such as enzymes, antibodies, or aptamers, can be immobilized on the electrode surface to selectively capture and react with the target biomarkers selectively.^239^ It is possible to employ various surface modification strategies, including covalent attachment, physical adsorption, or encapsulation within a polymer matrix, to optimize the immobilization the recognition elements and enhance the electrochemical performance of the device.^240^

### 5.4 Integration with Electronics and Wireless Communication Systems

**Figure 4:**
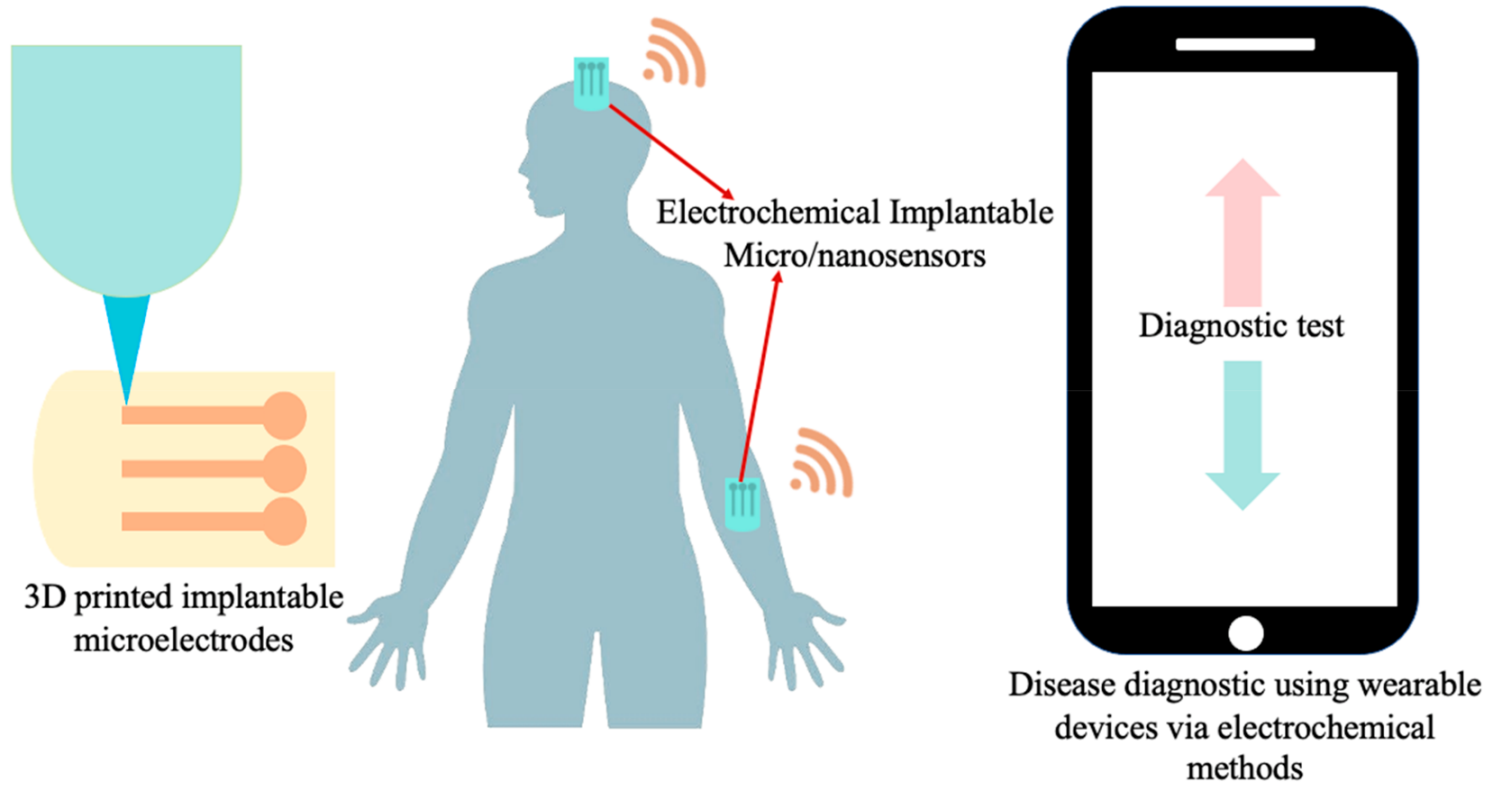
Disease diagnostic and continuous monitoring of cardiovascular and neurodegenerative disease.

Integrating 3D-printed implantable microelectrodes with electronics and wireless communication systems is crucial for enabling real-time in vivo monitoring of biomarker levels (Figure 4). Advanced miniaturized electronics can be incorporated into the device design, facilitating signal processing, amplification, and transmission to external data acquisition systems.^241^ The development of low-power, wireless communication protocols, such as bluetooth low energy (BLE) or near-field communication (NFC), allows for the seamless transmission of data to smartphones, wearable devices, or medical monitoring systems, providing healthcare providers with continuous, remote access to patient information.^242−244^

### 5.5 Longevity of Implantable Microelectrodes and Customizations

The longevity of 3D-printed implantable microelectrodes can vary based on material choice, device design, and the physiological environment to which they are exposed. Generally, the goal is to achieve long-term stability and functionality. Researchers and engineers work to select materials that offer durability, biocompatibility, and resistance to degradation over time.^245^ However, the specific lifespan of 3D-printed implantable microelectrodes can vary and depends on ongoing research and development to ensure their performance over extended periods within the body.

One of the notable advantages of 3D-printed implantable microelectrodes is their customization potential. These microelectrodes can be tailored for specific applications or patient needs due to the inherent flexibility of 3D printing technology. Researchers can design microelectrodes with intricate shapes, sizes, and functionalities to match individual anatomies and requirements. This level of customization enhances compatibility with the surrounding tissues, promotes integration, and minimizes foreign body reactions. Whether adjusting electrode sizes, incorporating sensors, or integrating drug delivery channels, 3D printing allows for versatile and patient-specific designs.^246^ As technology advances, combining customizable design and enhanced material properties in 3D printing continues to unlock new possibilities for developing implantable microelectrodes with extended lifespans and optimized functionality.

### 5.6 Integration of Multiple Functionalities

Integrating multiple functionalities, such as sensing and stimulation, into a single 3D-printed microelectrode device poses significant challenges. Coordinating diverse functions within a confined space demands precise engineering and innovative solutions. Ensuring minimal interference between functionalities is essential to maintain the performance and accuracy. Balancing electrical, mechanical, and thermal considerations becomes complex, as each functionality may have different requirements. Additionally, optimizing material selection is crucial, as it impacts the performance of individual functions and overall device biocompatibility. Coaxing various sensors, stimulators, and associated circuitry to work synergistically necessitates intricate design and sophisticated fabrication techniques. Moreover, miniaturization, crucial for implantable devices, intensifies the challenge of accommodating complicated features.^247,248^ Overcoming these challenges requires interdisciplinary collaboration, advanced manufacturing techniques, and meticulous testing to guarantee that integrated functionalities harmonize seamlessly, resulting in a reliable and efficient 3D-printed microelectrode device.

## 6 Applications of 3D-Printed Implantable Microelectrodes in Cardiovascular and Neurodegenerative Disease Diagnosis

### 6.1 Cardiovascular Disease Detection

**Figure 5:**
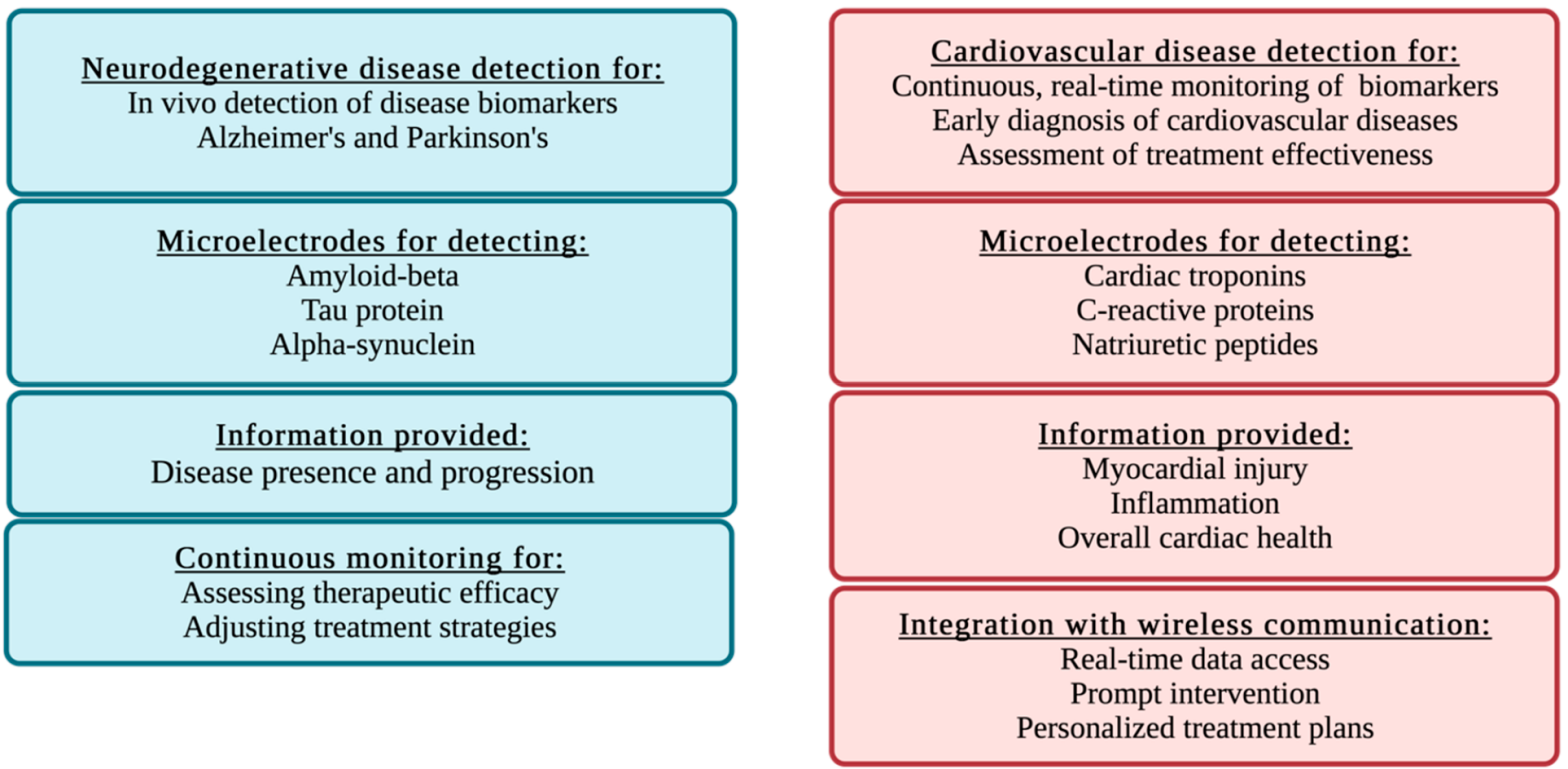
Applications and benefits of 3D-printed implantable microelectrodes for cardiovascular and neurodegenerative disease detection.

3D-printed implantable microelectrodes can be employed for the continuous, real-time monitoring of cardiac biomarkers, enabling the early diagnosis of cardiovascular diseases and the assessment of treatment effectiveness.^249^ For example, microelectrodes that detect cardiac troponins or C-reactive proteins can provide valuable information about myocardial injury, inflammation, and overall cardiac health. By integrating these devices with wireless communication systems, patients and healthcare providers can access real-time data on cardiac biomarker levels, facilitating prompt intervention and personalized treatment plans.^250^ Methods that enable the sensitive and label-free detection of protein biomarkers expanded the detection scope by utilizing phytic acid-doped polyaniline as a novel redox-charging polymer support, allowing the reagentless assaying of C-reactive protein in serum with spiked albumin with good sensitivity (Figure 5).^251^

### 6.2 Neurodegenerative Disease Detection

Similarly, 3D-printed implantable microelectrodes can be used for the in vivo detection of biomarkers associated with neurodegenerative diseases, such as Alzheimer’s and Parkinson’s. These devices can offer valuable insights into disease presence and progression by targeting specific biomarkers such as amyloid beta, tau protein, or alpha-synuclein. The continuous monitoring of biomarker levels in the brain can help clinicians assess the efficacy of therapeutic interventions and adjust treatment strategies accordingly (Figure 5).^252^

In conclusion, 3D-printed implantable microelectrodes for electrochemical detection of biomarkers are promising to improve the early diagnosis of cardiovascular and neurodegenerative diseases. By addressing critical design considerations, optimizing electrochemical performance, and integrating these devices with advanced electronics and wireless communication systems, researchers can develop innovative diagnostic tools that enable real-time, personalized patient care.^253^ Continued interdisciplinary collaboration, standardization, and ethical considerations will be essential in driving this technology’s successful development and clinical translation, ultimately contributing to enhanced patient outcomes and the advancement of precision medicine.

## 7 Challenges and Future Perspectives in the Development of 3D-Printed Implantable Microelectrodes

### 7.1 Biocompatibility, Long-Term Stability, Reliability, and Biodegradation

One of the most important challenges for implantable microelectrodes is their biocompatibility. Implantable microelectrodes must be biocompatible to avoid triggering an inflammatory response from the immune system, which can cause tissue damage and reduce the effectiveness of the electrodes. 3D printing materials, such as polymers and metals, may not be biocompatible, and their degradation products may cause toxicity. Therefore, developing biocompatible materials and coatings for 3D-printed microelectrodes is essential.^254,255^

**Figure 6:**
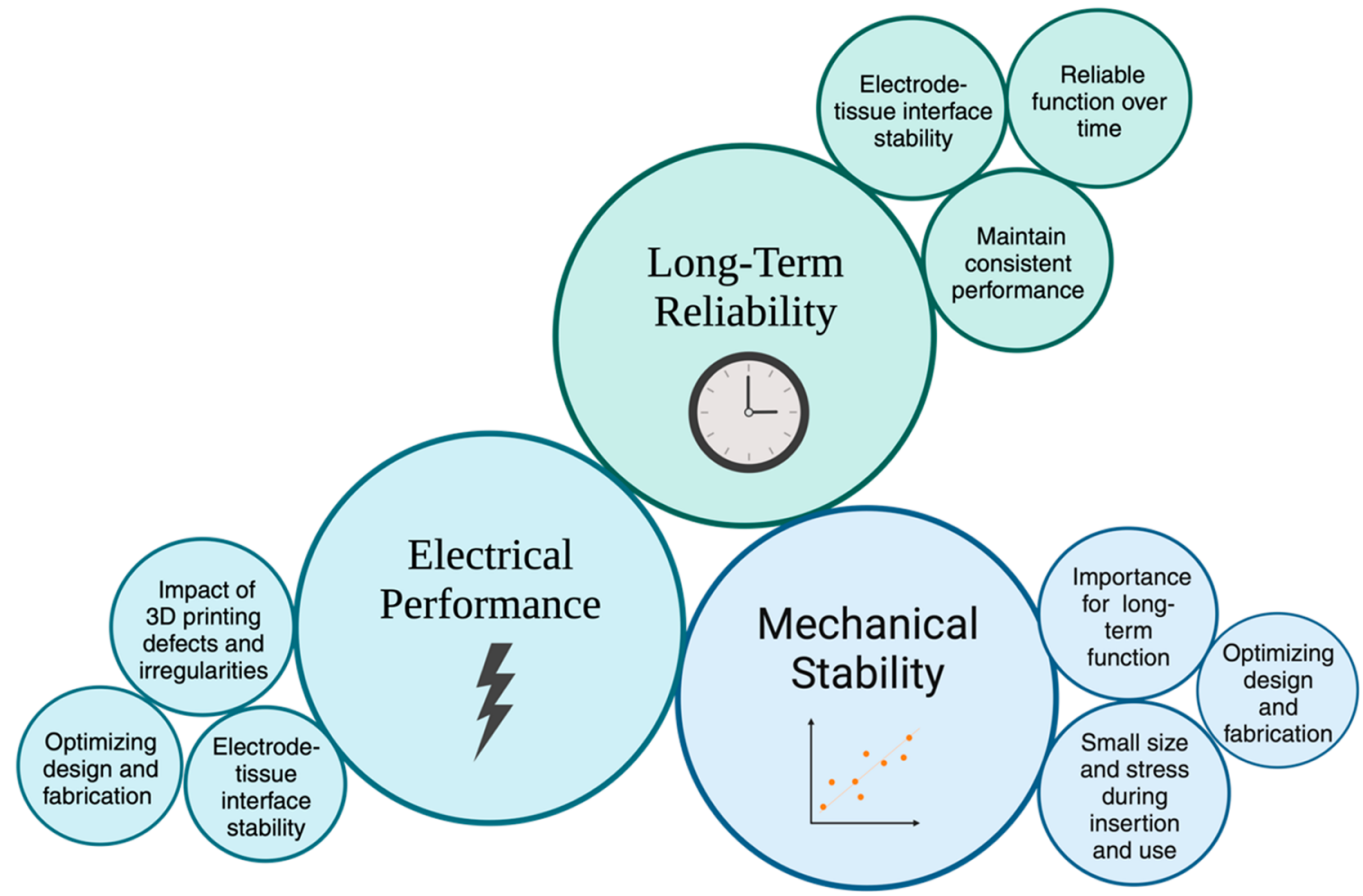
Visual representation of the challenges associated with implantable microelectrodes, focusing on long-term stability, reliability, and electrical performance.

Implantable microelectrodes must also be mechanically stable to maintain their function over an extended period. 3D printing can produce highly precise microelectrodes, but they may be prone to mechanical failure due to their small size and the stress they experience during insertion and use. This can cause the breakdown of electrodes or the electrodes to become dislodged, reducing their effectiveness. Therefore, optimizing the design and fabrication process is necessary to improve the mechanical stability of 3D-printed microelectrodes.^238^ Personalized diagnostics and treatment using 3D-printed implantable microelectrodes also face challenges related to long-term reliability (Figure 6). The microelectrodes need to function reliably over an extended period, which can be challenging, given the harsh environment of the human body. Factors such as corrosion, biofouling, and mechanical stress can all affect the long-term reliability of microelectrodes.^256,257^

The electrical performance of implantable microelectrodes is critical for their use in diagnostics and monitoring. However, 3D printing can introduce defects and irregularities in the electrode structure, affecting the electrical performance. Moreover, the long-term stability of the electrode–tissue interface can also be compromised due to factors such as corrosion and tissue encapsulation, leading to a decrease in the electrode’s electrical performance. Therefore, optimizing the electrode design and fabrication process is essential to ensure consistent electrical performance over an extended period (Figure 6).^258,259^

Another challenge for 3D-printed implantable microelectrodes is their biodegradation. Some 3D printing materials, such as polymers, may degrade over time, releasing toxic degradation products that can damage the surrounding tissue. Therefore, it is necessary to develop 3D printing materials that are stable and biocompatible over an extended period.^260^ Implantable microelectrodes must be sterilized before use to prevent infection. However, some 3D printing materials, such as gamma irradiation or ethylene oxide gas, may not withstand standard sterilization methods (Figure 6). Therefore, optimizing the design and fabrication process is essential to ensure that 3D-printed microelectrodes can be effectively sterilized without compromising their performance.^261,262^

### 7.2 Regulatory and Ethical Considerations

Regulatory and ethical considerations must be addressed as implantable microelectrodes progress to clinical implementation. The devices must meet stringent safety, efficacy, and quality regulatory requirements, necessitating close collaboration among researchers, industry partners, and regulatory agencies. Regulatory approval is one of the main challenges associated with personalized diagnostics and treatment using 3D-printed implantable microelectrodes. The development and license of medical devices is lengthy and expensive, requiring extensive testing and clinical trials. In addition, personalized medical devices may require additional regulatory scrutiny to ensure their safety and effectiveness.^263,264^ Personalized diagnostics and treatment using 3D-printed implantable microelectrodes raise ethical considerations. Patients may have concerns about the use of their data in the development of personalized medical devices. In addition, there may be questions about the accessibility and affordability of personalized medical devices, particularly for patients in low-income countries.^265,266^ Additionally, ethical concerns related to data privacy, informed consent, and potential misuse of the technology should be carefully considered and addressed to ensure the responsible development and deployment of these devices.^267^

### 7.3 Manufacturing Consistency

Manufacturing consistency is another challenge associated with personalized diagnostics and treatment using 3D-printed implantable microelectrodes. 3D printing technology can produce complex and patient-specific designs, but ensuring consistent quality across multiple devices can be challenging. Variations in the manufacturing process can affect the performance and reliability of the microelectrodes.^268,269^

### 7.4 Miniaturization and Integration of Microelectrode Systems

Implantable microelectrodes have great potential for diagnosing and monitoring cardiovascular and neurodegenerative diseases. With the advent of 3D printing technology, producing microelectrodes with high precision and accuracy is now possible. However, several challenges need to be addressed to achieve successful miniaturization of 3D-printed implantable microelectrodes. The electrode size is critical for accurate monitoring and diagnosis and for minimizing the implant’s invasiveness. The current trend is to make the electrodes as small as possible, which requires advanced manufacturing techniques to achieve high precision and accuracy. Another challenge is the integration of microelectronics and sensors into the electrode. This requires advanced microfabrication techniques that integrate multiple components into a single device.

Additionally, using biocompatible materials is essential for the long-term success of the implant. There are also challenges related to the implantation process itself. Implantable microelectrodes must be inserted into the body with minimal invasiveness to avoid tissue damage and inflammation. This requires specialized surgical techniques and tools to insert the electrode precisely and with minimal tissue disruption.^270−272^

Despite these challenges, there have been significant advances in 3D-printed implantable microelectrodes. For example, researchers have successfully created implantable microelectrodes with diameters as small as submicrometres to several nanometer dimensions using 3D printing techniques.^273,274^ Other researchers have developed microelectrode arrays with integrated microelectronics and sensors for real-time monitoring of neural activity. In conclusion, miniaturizing 3D-printed implantable microelectrodes for cardiovascular and neurodegenerative disease diagnostics and monitoring presents several challenges, including the miniaturization of the electrodes, the integration of microelectronics and sensors, and the implantation process. However, with continued research and development, 3D-printed implantable microelectrodes have the potential to revolutionize the diagnosis and treatment of these diseases.

## 8 Prospects of 3D-Printed Microelectrodes in Diagnosis

**Figure 7:**
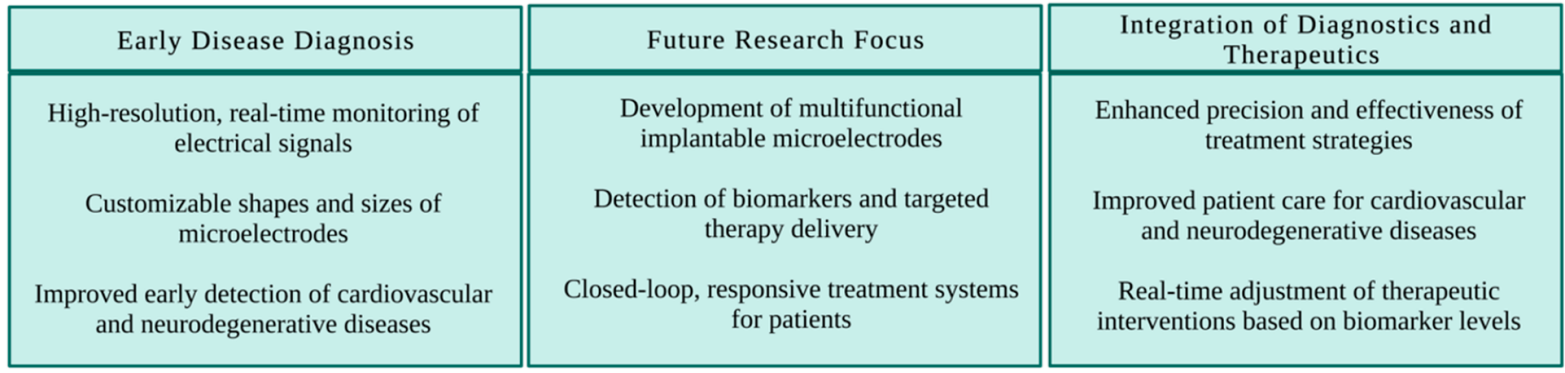
Summary of the potential of 3D-printed microelectrodes in diagnosis, highlighting their role in early disease diagnosis, the focus of future research, and the integration of diagnostic and therapeutic capabilities.

3D-printed microelectrodes can potentially improve early diagnosis of cardiovascular and neurodegenerative diseases by providing high-resolution, real-time monitoring of electrical signals in the body. The ability to produce microelectrodes with customizable shapes and sizes, combined with electronic read-out circuitry, allows for high-resolution, real-time monitoring of electrical signals in the body (Figure 7). This can enable early disease detection, leading to early intervention and treatment and improved patient outcomes.^275−278^

Future research could focus on developing multifunctional implantable microelectrodes that detect biomarkers and deliver targeted therapies based on detected biomarker levels. Such devices would enable closed-loop, responsive treatment systems that adjust therapeutic interventions in real-time according to patient needs.^279^ This integration of diagnostic and therapeutic capabilities could significantly enhance the precision and effectiveness of treatment strategies for cardiovascular and neurodegenerative diseases.

The potential of 3D-printed microelectrodes to revolutionize early diagnosis lies in their ability to provide unprecedented resolution and sensitivity in monitoring and analyzing neural activity. Traditional microelectrodes are usually made from metal wires or glass pipets and have limited flexibility and control over their geometries. In contrast, 3D printing allows for fabricating intricate microelectrode structures with sub-micrometer precision, enabling researchers to customize the electrodes’ size, shape, and material properties to suit specific experimental requirements. Furthermore, 3D printing can integrate microelectrodes with other functional components, such as microfluidic channels, sensors, and actuators, creating highly integrated and multifunctional devices for real-time monitoring and manipulation of biological systems (Figure 7).

Several recent studies have demonstrated the potential of 3D-printed microelectrodes in the early diagnosis of neurological disorders. For instance, researchers have used 3D printing to create microelectrodes/nanoelectrodes with various shapes and sizes and tested their performance in detecting dopamine release in live brain tissue.^280^ The results showed that the 3D-printed microelectrodes could detect dopamine signals with high sensitivity and selectivity, providing a powerful tool for studying the mechanisms of PD and other related disorders.^281,282^ Similarly, researchers have developed a 3D-printed microelectrode array for high-resolution mapping of epileptic activity in mice brains. The array consisted of 16 independently addressable microelectrodes with a total size of less than 1 mm^2^ and could detect submillisecond changes in neural activity. The researchers demonstrated that the array could accurately localize the sources of epileptic activity in the brain and provide insights into the underlying mechanisms of epilepsy.^283,284^

Recent developments in machine learning and artificial intelligence can significantly improve the performance and reliability of these devices.^285^ One potential application of machine learning is optimizing the design, process parameters, and material properties required for specific needs and fabrication of microelectrodes. Machine learning algorithms can analyze large data sets of microelectrode fabrication parameters and performance metrics to identify the optimal set of parameters for achieving high sensitivity and selectivity. This can significantly reduce the time and cost required for the iterative fabrication and testing of microelectrodes. Another application of machine learning is in developing algorithms for real-time and in situ signal processing and analysis of implanted microelectrode data.^286^ Machine learning algorithms can be trained to recognize microelectrode signal patterns specific to different cardiovascular and neurodegenerative diseases such as arrhythmia or heart failure. This can enable real-time monitoring of cardiovascular health and early disease detection, allowing for timely intervention and treatment (Figure 7).

In conclusion, 3D printing technology can potentially revolutionize the field of microelectrodes and the early diagnosis of neurological disorders and other diseases. The ability to fabricate highly customized and multifunctional microelectrodes with sub-micrometer precision and material diversity can significantly enhance our understanding of complex biological systems and pave the way for developing new diagnostic and therapeutic approaches. However, further research is needed to optimize the design, fabrication, and integration of 3D-printed microelectrodes and to explore their potential applications in clinical settings.^287^
